# DGAT1 Expression Promotes Ovarian Cancer Progression and Is Associated with Poor Prognosis

**DOI:** 10.1155/2021/6636791

**Published:** 2021-05-14

**Authors:** Leilei Xia, Ye Wang, Shengyun Cai, Mingjuan Xu

**Affiliations:** ^1^Department of Obstetrics and Gynecology, Changhai Hospital, Navy Medical University, Shanghai, China; ^2^Department of Urology, Chinese People's Liberation Army(PLA) General Hospital/PLA Medical School, Beijing, China

## Abstract

**Background:**

Ovarian cancer is the most fatal gynecological malignancy. Owing to its insidious onset, rapid development, and poor prognosis, ovarian cancer is the fifth most common cause of death in women. Although immunotherapy-related drugs, such as Olaparib, can alleviate ovarian cancer progression, there are no remarkable breakthroughs for its effective treatment. It is considered that the transformation of normal cells to cancerous ones involves “recoding” of certain metabolic pathways. Diacylglycerol O-acyltransferase 1 (DGAT1) can synthesize triglycerides by transferring acyl-CoA to diacylglycerol, which plays a key role in lipid synthesis. However, the role of DGAT1 in ovarian cancer is not yet elucidated.

**Materials and Methods:**

We analyzed the correlation between DGAT1 and ovarian cancer staging, grading, vascular invasion, and prognosis by collating the information of ovarian cancer specimens from The Cancer Genome Atlas (TCGA) database. Furthermore, the effects of DGAT1 expression on proliferation, migration, invasion, and tumor growth were studied using ovarian cancer cell lines. GSEA was used to analyze the KEGG pathways and biological function enriched because of DGAT1 expression in ovarian cancer.

**Results:**

The expression of DGAT1 was elevated in advanced (*p* = 0.0432), poorly differentiated (*p* = 0.0148), and vascular invaded (*p* = 0.0002) ovarian cancer specimens. Prognosis among patients with high expression of DGAT1 was poor. After DGAT1 expression was interfered, proliferation, migration, invasion, colony forming, and tumor growth of ovarian cancer cells were inhibited. In addition, GSEA showed that DGAT1 may be involved in the immune process.

**Conclusion:**

DGAT1 expression is associated with the clinical phenotype of ovarian cancer. We suggest that DGAT1 has potential implications in the treatment of ovarian cancer.

## 1. Background

Ovarian cancer is the most fatal of all gynecological malignancies [1]. Since most patients are diagnosed with ovarian cancer at the advanced stage of the disorder, the mortality rate remains high. It is estimated that more than 100,000 women die of ovarian cancer annually worldwide. Due to its insidious onset, rapid development, and poor prognosis, it is the fifth most common cause of death in women [2]. It is hypothesized that the transition of normal cells to cancerous cells involves a “recoding” of metabolic pathways [3, 4], including but not limited to regulation of glycolysis [5, 6]. At present, there are hypotheses stating that cancer cells promote de novo synthesis of lipids or develop into “fat phenotypes” [7], which play an important role in tumor progression. Moreover, ovary is an important organ for hormone production, wherein the precursors are lipid molecules. The ovary, hormone production, and lipid synthesis are closely entangled with each other. Therefore, elucidating the role and mechanism of lipid metabolism dysfunction in ovarian cancer may provide new insights for the diagnosis and treatment of ovarian cancer. Diacylglycerol o-acyltransferase 1 (DGAT1) is a transmembrane protein expressed in the endoplasmic reticulum. It synthesizes triglycerides by transferring acyl-CoA to diacylglycerol, which plays a key role in the lipid synthesis pathway [8, 9]. However, the role of DGAT1 in ovarian cancer remains poorly understood.

## 2. Materials and Methods

### 2.1. Bioinformatics Data Mining

We used Genomic Data Commons (GDC) software to download ovarian cancer (OV) RNA-Seq data from The Cancer Genome Atlas (TCGA) dataset in FPKM format. Clinical information of patients with OV was retrieved, followed by extraction of information on the clinical stage, pathological grade, vascular invasion of cancer, and survival information of the patients. Postoperative death time or last follow-up time was considered as the survival time of patients. Gene set enrichment analysis (GSEA) was performed using the program's Java software available on the website (https://www.gsea-msigdb.org/gsea/index.jsp). GEPIA website was used to evaluate the expression of DGAT1 in ovarian cancer and normal tissues (http://gepia.cancer-pku.cn/). TIMER website was utilized to analyze the correlation between DGAT1 and immune cells (https://cistrome.shinyapps.io/timer/).

### 2.2. Cell Culture

Ovarian carcinoma cell lines (PEO4, PEO1, OVCAR-5, IGROV1, OVCAR-8, ES2, SKOV3, MCAS, and OVCAR3) were appropriately maintained at Shanghai Cancer Institute, Shanghai Jiao Tong University. Cells were cultured in RPMI-1640 medium containing 10% FBS, penicillin (100 units/ml), and streptomycin (100 *μ*g/ml). All cells were maintained in a humidified CO_2_ (5%) incubator at 37°C.

### 2.3. Real-Time PCR

RNA was extracted using TRIzol (Takara, Japan) and reverse transcribed to cDNA using PrimeScript RT-PCR kit (Takara, Japan). Then, real-time PCR was performed using SYBR Premix Ex Taq (Takara, Japan) by Applied Biosystems 7500 Real-time PCR. Relative expression of DGAT1 was evaluated against normalized GAPDH. Primer sequences used for this assay are displayed in Table 1.

### 2.4. Western Blotting Analysis

Total protein was extracted from DGAT1 and PEO4 cells using buffer with proteinase inhibitor cocktail and phosphatase inhibitor cocktail. Then, proteins were electrophoresed in 10% SDS/PAGE gels and transferred to polyvinylidene fluoride membranes. After blocking with BSA for 2 h, incubating with the DGAT1 or *β*-actin primary antibodies at 1 : 1000 at 4°C overnight, washing with T-BST three times, and incubating with HRP-conjugated secondary antibodies for 2 h at room temperature, the membranes were visualized with ECL Western blotting detection reagents (Pierce Biotechnology, Rockford).

### 2.5. DGAT1 Knockdown and Cell Transfection

Lentiviral constructs (sh-Ctrl and sh-DGAT1) were purchased from OBiO Technology Corp., Ltd. (Shanghai, China). OVCAR-5 and PEO4 cells were transfected with sh-Ctrl or sh-DGAT1 with the supplementation of 6 *μ*g/ml polybrene. Transfected cells were cultured in RPMI-1640 medium supplemented with 2 *μ*g/ml puromycin to filter stable cells.

### 2.6. CCK-8 Cell Viability Assay

OVCAR-5 and PEO4 cells transfected with sh-Ctrl and sh-DGAT1 were seeded in 96-well plates at a density of 2000 cells/well. Then, CCK-8 (Dojindo Molecular Technologies, Japan) was added at 0, 1, 2, 3, and 4 days postseeding according to the manufacturer's protocol. The plates were incubated in a humidified CO_2_ (5%) incubator at 37°C for 1 hour. Cell viability was determined by measuring absorbance at 450 nm using Power Wave XS microplate reader (BioTek, USA).

### 2.7. Colony Formation Assay

Transfected OVCAR-5 and PEO4 cells were cultured in 6-well plates at 1,000 cells/well. After 2 weeks of incubation, the clones were fixed, stained with 0.1% (*w*/*v*) crystal violet solution, and scanned using HP scanner.

### 2.8. Tumor Cell Migration (Transwell) Assay

OVCAR-5 and PEO4 cells transfected with sh-Ctrl and sh-DGAT1 were seeded in the upper chamber at a density of 5 × 10^4^/ml in 200 *μ*l of serum-free RPMI-1640 medium, whereas 700 *μ*l of medium containing 20% FBS was added in the lower chamber. After incubating for 24 hours, cells were fixed and stained with 0.1% (*w*/*v*) crystal violet solution. Image(s) were captured and cells were counted at three random regions.

### 2.9. Mouse Xenograft Model

Athymic female nu/nu mice (6-week-old) were purchased and fed at East China Normal University. OVCAR-5 cells transfected with sh-Ctrl and sh-DGAT1 were injected in subcutaneous tissue of nude mice. Forty days later, mice were euthanized and the tumors were weighed.

### 2.10. Statistical Analysis

Statistical analyses were performed using GraphPad 7.0. Differences between two groups were estimated by two-tailed Student's *t*-tests. Kaplan–Meier plotter was used for survival analysis; a *p* value of less than 0.05 was considered statistically significant.

## 3. Results

### 3.1. DGAT1 Overexpression Is Associated with Poor Survival in Ovarian Cancer

**Figure 1:**
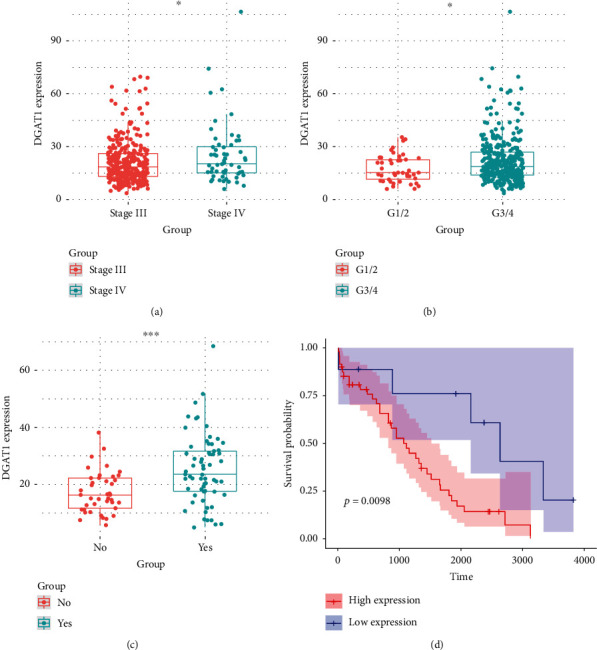
Upregulation of DGAT1 expression is associated with poor survival in ovarian cancer. (a) DGAT1 expression is upregulated in stage IV ovarian cancer as compared to stage III. (b) DGAT1 expression is upregulated in histological grade 3/4 as compared to histological grade 1/2. (c) DGAT1 is overexpressed in patients with venous invasion as compared to those without venous invasion. (d) Patients in stage IV with higher expression of DGAT1 have an unfavorable prognosis as compared to those with lower expression of DGAT1 (*p* ≤ .05 = ∗, *p* ≤ .001 = ∗∗∗).

DGAT1 expression levels were obtained from the TCGA database to analyze its expression in different phenotypes. DGAT1 was significantly overexpressed in stage IV cancer tissues compared to the stage III specimens (Figure 1(a)). Furthermore, DGAT1 was highly expressed in histological grade 3/4 cancer tissues compared to that in histological grade 1/2 (Figure 1(b)). Additionally, its expression levels were higher in patients with venous invasion than in those without venous invasion (Figure 1(c)). Interestingly, patients in stage IV with higher expression of DGAT1 had an unfavorable prognosis as compared to those with lower expression of DGAT1 (Figure 1(d)). These results give us a hint that DGAT1 may serve as a crucial gene in the development of ovarian cancer. Then, we test the expression of DGAT1 between cancer tissues and normal tissues; furthermore, as shown in Supplement Figure 1, DGAT1 expression has a downward trend but has no statistical difference between these groups.

### 3.2. DGAT1 Promotes Proliferation, Colonization, and Metastasis of Ovarian Cancer

**Figure 2:**
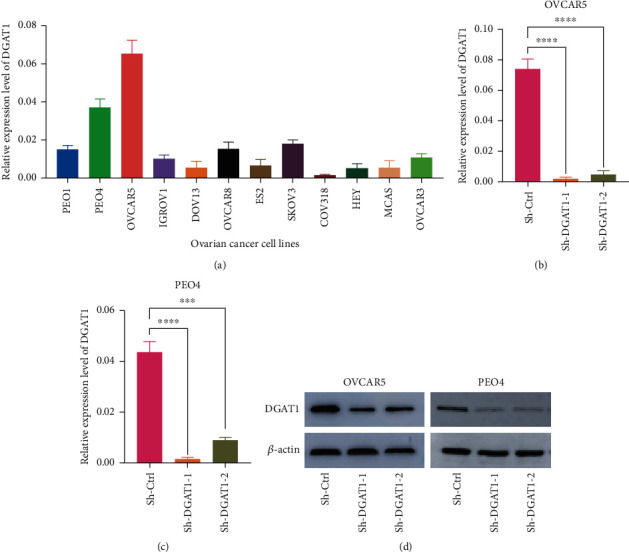
DGAT1 is overexpressed in OVCAR-5 and PEO4 cells. (a) DGAT1 mRNA expression in ovarian cancer cell lines. (b and c) qRT-PCR analysis of knockdown efficiency of DGAT1 in OVCAR-5 and PEO4 cells. (d) Western blot tests the knockdown efficiency of DGAT1 in OVCAR-5 and PEO4 cells (*p* ≤ .001 = ∗∗∗, *p* ≤ .0001 = ∗∗∗∗).

**Figure 3:**
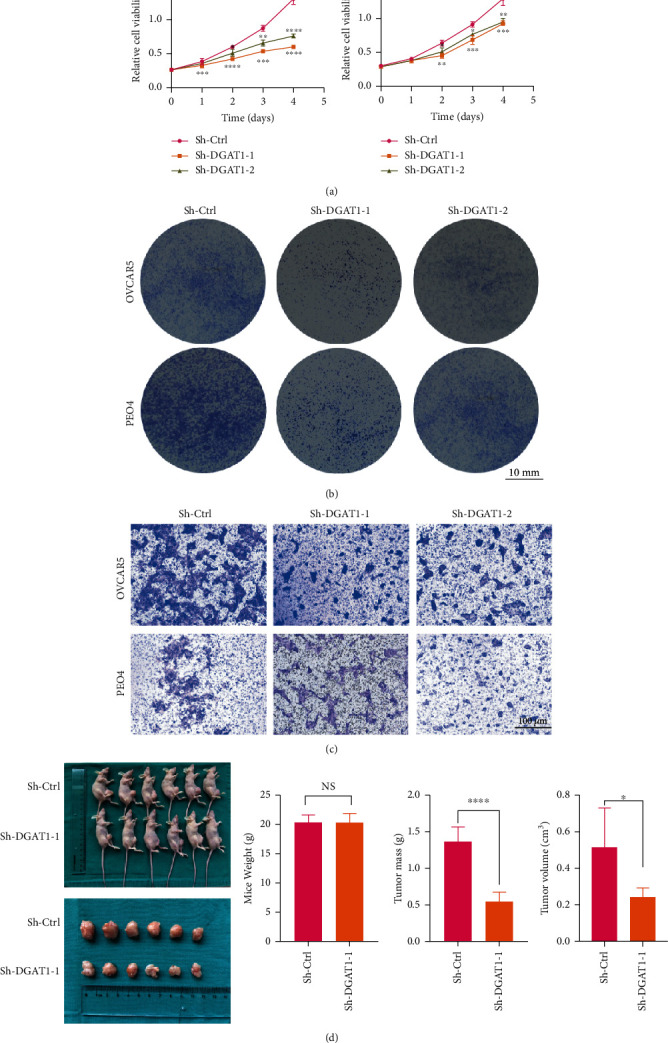
DGAT1 promotes proliferation, colonization, and metastasis of ovarian cancer. (a) CCK-8 cell proliferation assay was carried out using OVCAR-5 and PEO4 cells with sh-Ctrl, sh-DGAT1. (b) Colony formation assay was performed using OVCAR-5 and PEO4 cells with sh-Ctrl, sh-DGAT1. (c) Transwell migration assay was conducted using OVCAR-5 and PEO4 cells with sh-Ctrl, sh-DGAT1. (d) Tumor model is established using sh-Ctrl, sh-DGAT1 OVCAR-5 transfected cells (*p* ≥ .05 = ns, *p* ≤ .05 = ∗, *p* ≤ .01 = ∗∗, *p* ≤ .001 = ∗∗∗, *p* ≤ .0001 = ∗∗∗∗).

According to the results of the quantitative RT-PCR analysis, DGAT1 was highly expressed in OVCAR-5 and PEO4 cells (Figure 2(a)). Moreover, both RT-PCR and western blot analysis showed that DGAT1 was stably knocked down in both these cell lines (Figures 2(b)–2(d)). Due to bioinformatics analysis showed DGAT1 has a close relationship with ovarian cancer phenotype, and we tested DGAT1 function in ovarian cell lines on the proliferation and metastasis. As shown in Figure 3, proliferation, colonization ability, and migration ability of cells with knocked down DGAT1 were significantly reduced in vitro (Figures 3(a)–3(c)). To further elucidate the role of DGAT1 in ovarian cancer, subcutaneous tumor-bearing model was established. As shown in Figures 3(d)–3(e), tumor weight and volume were significantly decreased when DGAT1 expression was suppressed. Thus, our findings suggest that DGAT1 is involved in the development and metastasis of ovarian cancer.

### 3.3. DGAT1 Expression Alters the Cell Cycle, Adherens Junction, and Immune Process in Ovarian Cancer

**Figure 4:**
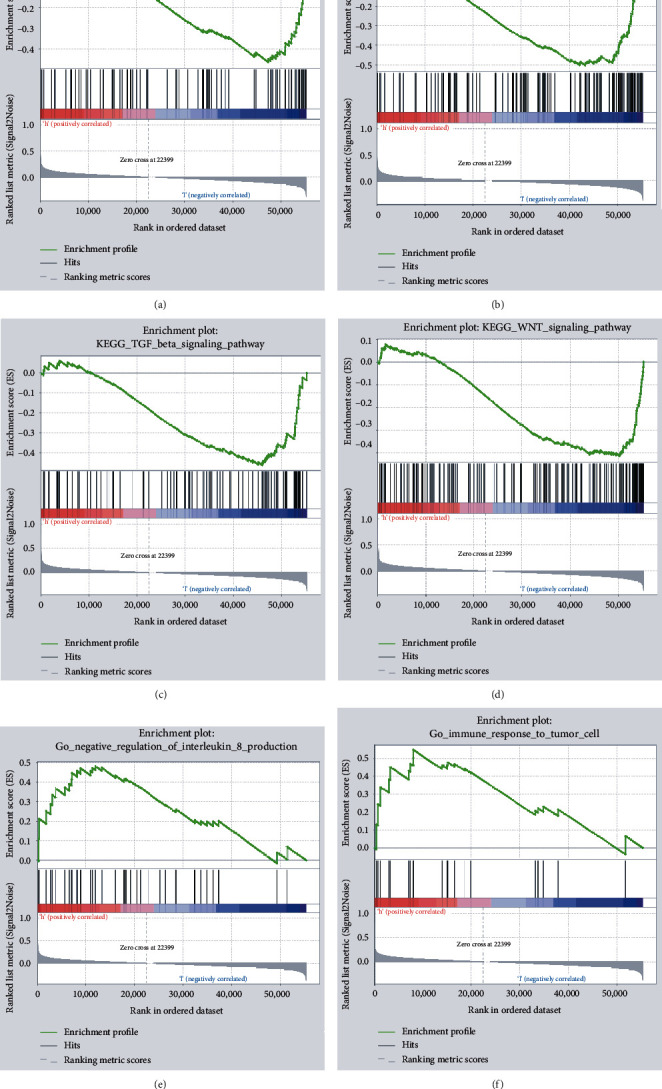
GSEA of KEGG pathways and biological function of DGAT1 involved in ovarian cancer. GSEA of samples with high and low expressions of DGAT1 based on TCGA datasets.

GSEA was carried out to determine the KEGG pathways and biological function of DGAT1 expression in ovarian cancer. Based on DGAT1 expression levels in the ovarian cancer, samples of TCGA datasets were categorized into two groups, namely, DGAT1-low group (<11.36) and DGAT1-high group (≥11.36). As shown in Figure 4, adherens junction, cell cycle, TGF*β* signaling pathway, Wnt signaling pathway, immune response to tumor cell, and negative regulation of IL-8 production were altered. As the immune system plays an irreplaceable role in the development of cancer, thus, TIMER website was utilized to explore the correlation between DGAT1 and immune cells in ovarian cancer. We found that DGAT1 has a significant relation with B cells (*p* = 0.0373).

## 4. Discussion

We found that DGAT1 expression is associated with ovarian cancer. The expression of DGAT1 was higher in advanced (*p* = 0.0432), poorly differentiated (*p* = 0.0148), and vascular invaded (*p* = 0.0002) ovarian cancer specimens. The prognosis of ovarian cancer among patients with high expression of DGAT1 was poor. After the expression of DGAT1 was interfered, the proliferation, migration, invasion, colony forming, and tumor growth of ovarian cancer cells were inhibited.

The transformation of normal cells to cancer cells involves a “recoding” of metabolic pathways [3, 4], including the regulation of glycolysis [5, 6], glutamine dependent replenishment [10–12], and lipid production [7, 11]. Although metabolic dysregulation is hypothesized to be one of the characteristics of cancer, how the metabolic dysregulation occurs and how the products of this dysregulation play a role in the occurrence and development of cancer remain unclear. Currently, it is assumed that the cancer cells promote de novo synthesis of lipids in the dysfunctional metabolic pathway. It has been developed into “fat phenotype” [7], wherein lipid synthesis plays an important role in tumor progression. Previous studies have shown that fatty acid synthase (FAS), an enzyme that synthesizes fatty acids from acetic acid and malonyl-CoA, is associated with poor prognosis among patients with breast cancer. Moreover, inhibition of FAS can significantly reduce cell proliferation and cell survival rate, thereby inhibiting tumor growth in vivo [7, 13, 14]. This suggests that FAS may promote tumor growth by providing metabolic substrate for energy production [15–17]. Similarly, monoacylglycerol lipase (MAGL) can promote the survival, migration, and tumorigenicity of tumor cells by regulating free fatty acid [18]. Moreover, other functions of lipid substances support their role in tumor progression and development. For example, most of the cell membrane is composed of lipids; an increased synthesis of lipids can accelerate the synthesis of cell membrane to adapt to the rapid proliferation of tumor cells [11]. Furthermore, lipids constitute the membrane of organelles that are involved in cell signal transduction and transport [19–21]. For instance, PI (3,4,5) P3, which is synthesized by phosphatidylinositol-3 kinase, promotes cell survival and proliferation by activating the protein kinase B/Akt pathway [22, 23]; lysophosphatidic acid (LPA), which transmits signals through G-protein coupled receptors, thereby promoting the invasion of cancer cells [24, 25]; furthermore, prostacyclin catalyzed by cyclooxygenase promotes tumor migration [26, 27].

DGAT1 is a multimembrane protein expressed in endoplasmic reticulum. It can synthesize triglycerides by transferring acyl-CoA to diacylglycerol, which plays a key role in lipid synthesis [8, 9]. In vitro, DGAT1 and mannosyl (alpha-1,3-)-glycoprotein beta-1,2-N-acetylglucosaminyltransferase (MGAT) can catalyze the direct conversion of monoglycerides to triglycerides [28]. Triglycerides play an important role in energy storage and utilization, as well as in membrane lipid synthesis. Although triglycerides are vital in normal physiological function, they can have adverse effects if synthesized excessively, which may cause obesity (in adipose tissue) and organ dysfunction (nonadipose tissue). It is suggested that DGAT1 participates in the synthesis of very low-density protein (VLDL) and is involved in obesity and insulin resistance [29, 30]. DGAT1 is a member of the MBOAT family, wherein other members of this family play an important role in lipid metabolism, signal transduction, and hormone treatment [31]. However, there are few studies reporting the biological function of DGAT1. It has been reported that the activity of DGAT1 can be significantly increased by increasing the concentration of glucose in the medium, whereas the activity of DGAT1 can be markedly inhibited when the concentration of glucose is reduced, indicating that the activity of DGAT1 is regulated by glucose concentration [32].

Moreover, the results of our GSEA suggest that DGAT1 is involved in the negative regulation of IL-8 production. Previously, it has been shown that the expression of IL-8 is significantly increased in ovarian cancer [33], highlighting the importance of this chemokine in the development of ovarian cancer. Moreover, IL-8 can promote the migration of human ovarian cancer cells through the Wnt/*β*-catenin pathway-mediated EMT [34]. Chemokines can affect tumor biology in several ways. On the one hand, some chemokines can stimulate the growth of tumor cells and promote angiogenesis, thereby promoting the tumor growth and metastasis. On the other hand, some chemokines can inhibit growth and metastasis of tumor by the chemotactic activation of the immune cells and inhibition of tumor angiogenesis. Recently, many studies have reported the effects of chemokines on tumor biology. Tumor cells and their related inflammatory cells can secrete a variety of chemokines, which are mainly secreted by autocrine or paracrine signaling. These chemokines can form a complex network that possibly affects the tumor microenvironment. Chemokines and their receptors play a vital role in biological behaviors of tumor growth, metastasis, and invasion. Hence, they are considered as one of the hotspots in tumor therapy and may be novel targets for cancer therapy.

GSEA showed that DGAT1 may participate in the Wnt signaling pathway. It has been demonstrated that the Wnt signaling pathway plays an important role in the embryonic development of ovarian tissue, as well as the proliferation, differentiation, and malignant transformation of ovarian cells. Additionally, changes in membrane, cytoplasm, nucleus, and proteins that affect the Wnt signaling pathway play an important role in the pathogenesis of ovarian cancer.

Therefore, DGAT1 is presumed to be a novel target for the treatment of ovarian cancer. However, the mechanism by which DGAT1 regulates the progression of ovarian cancer needs to be further studied.

## 5. Conclusion

DGAT1 is closely associated with the clinical phenotype of ovarian cancer. Therefore, we hypothesize that DGAT1 holds potential implications in the treatment of ovarian cancer.

## Figures and Tables

**Table 1:** Primers used in this study.

Primer	Sequence 5′-3′
DGAT1 forward	TGCCTTGCCCTCTGCTCTTCT
DGAT1 reverse	CCTGACCTCCCGCTACCATCAA
GAPDH forward	CTGGGCTACACTGAGCACC
GAPDH reverse	AAGTGGTCGTTGAGGGCAATG

## Data Availability

https://portal.gdc.cancer.gov/

## Supplementary Materials

Supplementary 1 Supplement Figure 1: DGAT1 expression has no statistically significant difference between ovarian cancer tissues and normal tissues.

Supplementary 2 Supplement Figure 2: DGAT1 expression in ovarian cancer has a significant correlation with B cells.
