# Supplementary figures and images for: DGAT1 Expression Promotes Ovarian Cancer Progression and Is Associated with Poor Prognosis
Source: J Immunol Res. 2021 May 14;2021:6636791. doi: 10.1155/2021/6636791 (PMC8141271; doi:10.1155/2021/6636791)

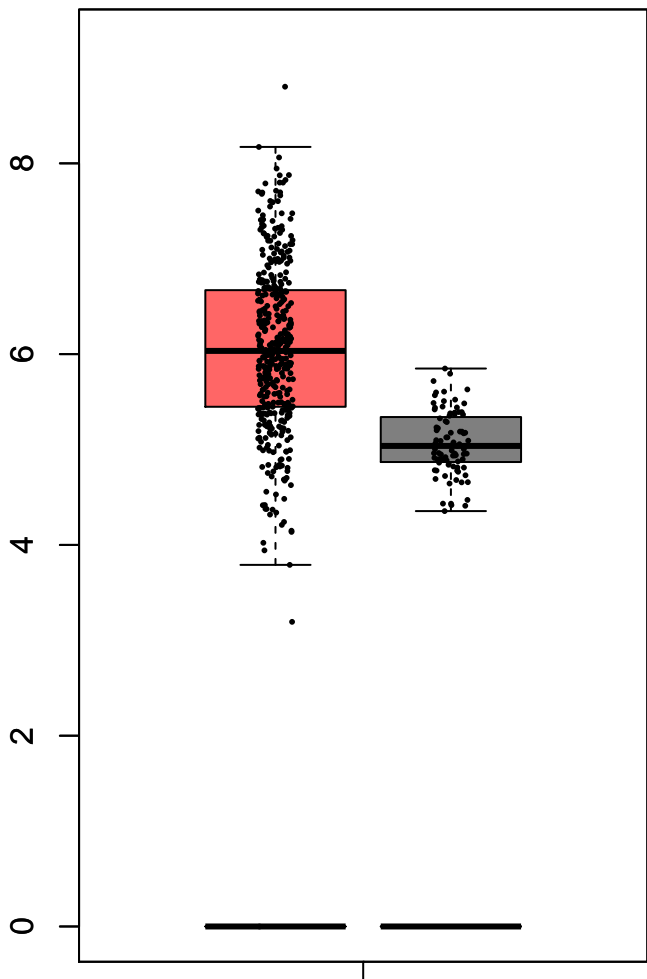

OV  
(num(T)=426; num(N)=88)

Supplement: Supplementary 1 — Supplement Figure 1: DGAT1 expression has no statistically significant difference between ovarian cancer tissues and normal tissues. [file 6636791.f1.pdf]

DGAT1 Expression Level (log2 TPM)

OV

Purity

Rho = -0.151  
p = 1.67e-02

0.4

0.6

0.8

1.0

Purity

B cell\_TIMER

Rho = 0.132  
p = 3.73e-02

0.0

0.2

0.4

0.6

Infiltration Level

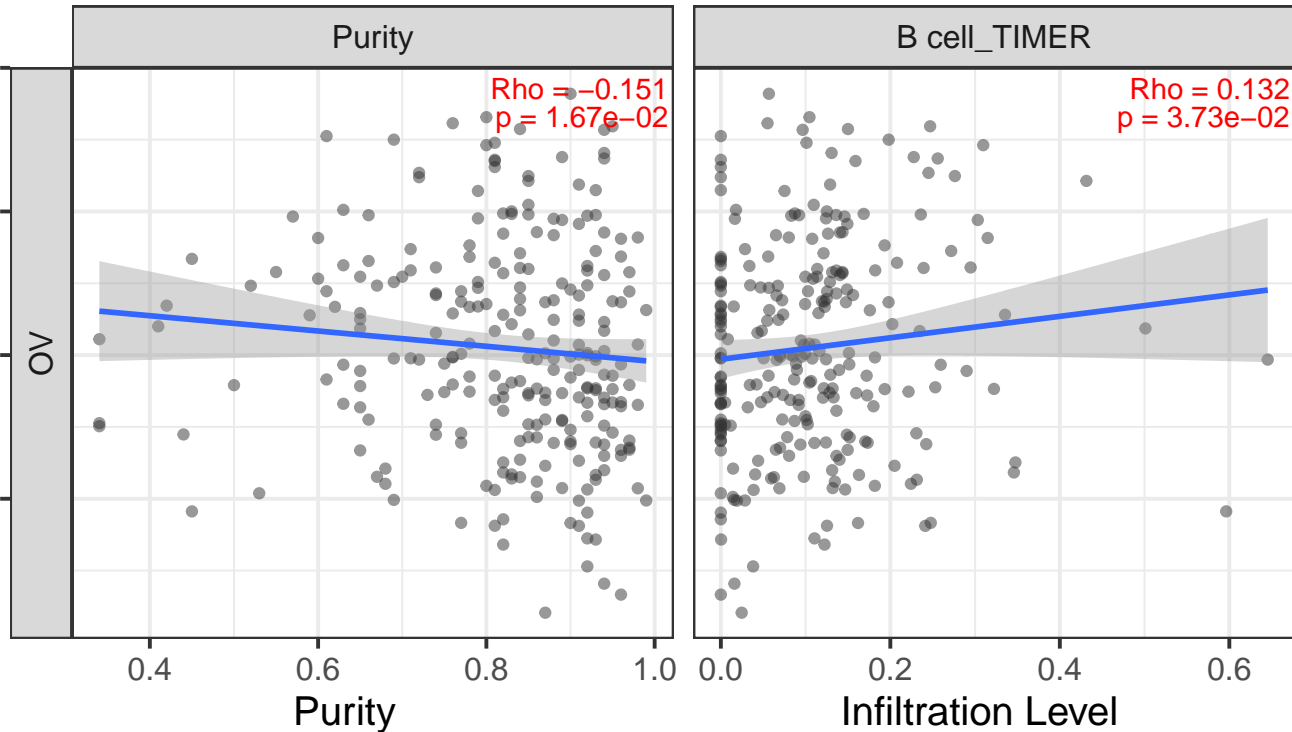

Supplement: Supplementary 2 — Supplement Figure 2: DGAT1 expression in ovarian cancer has a significant correlation with B cells. [file 6636791.f2.pdf]
